# First person – Amy Bongetti

**DOI:** 10.1242/dmm.053009

**Published:** 2026-06-01

## Abstract

First Person is a series of interviews with the first authors of a selection of papers published in Disease Models & Mechanisms, helping researchers promote themselves alongside their papers. Amy Bongetti is first author on ‘ Evaluating skeletal muscle dysfunction and recovery in a zymosan model of critical illness in mice’, published in DMM. Amy is an associate research fellow in the lab of Professor Gordon Lynch at The University of Melbourne, Victoria, Australia, investigating skeletal muscle wasting and weakness in critically ill patients, particularly in the refinement of animal models to improve mechanistic understanding of this condition and the translation of therapeutics from bench to bedside.

**Figure:**
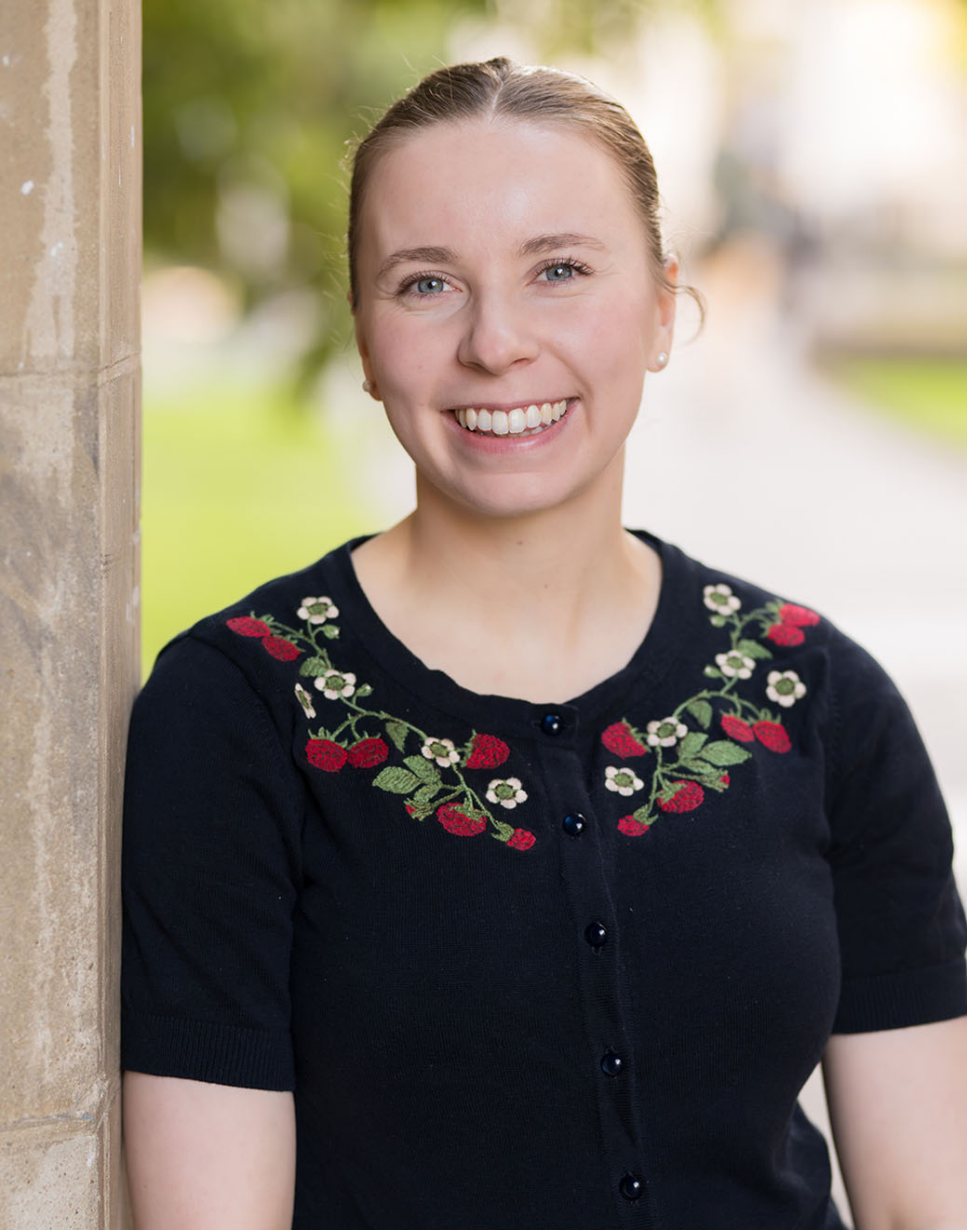
Amy Bongetti

**Who or what inspired you to become a scientist?**

I have always had a deep curiosity about the way the world works and how our body functions. A background in elite sport (karate) inspired a deeper interest in muscle physiology, which led me to undertake a research project during my undergraduate degree. This experience made me realise that research was my passion! During my graduate studies investigating the mechanisms underlying critical illness, I was fortunate to forge wonderful collaborations both at home and abroad, working on clinical trials with dietitians and intensivists and contributing to working groups devising treatments for intensive care unit (ICU)-acquired weakness. I am also a passionate educator and enjoy the creativity, scientific rigour and diverse range of people I work with as an academic.

**What is the main question or challenge in disease biology you are addressing in this paper? How did you go about investigating your question or challenge?**

My research aims to address gaps in understanding muscle dysfunction in patients after critical illness. A contributing factor to current limitations in understanding these functional impairments is the lack of comprehensive assessment using rigorous pre-clinical models. We investigated the zymosan-induced model of critical illness in mice, assessing whole-body metabolic and respiratory function, effects on muscle groups of varying fibre composition, and muscle function (strength and fatigue). We found that not all muscle groups respond to critical illness in the same way and can have different trajectories for wasting and recovery. These findings provide important insights into the mechanisms underlying functional recovery after critical illness with translational relevance to patients with ICU-acquired weakness.

**How would you explain the main findings of your paper to non-scientific family and friends?**

Up to half of all patients admitted to the ICU can experience muscle wasting and weakness, which can severely affect their strength and general health after discharge. The underlying reasons for this ICU-acquired muscle weakness are not understood completely, and there are currently no effective therapies in routine standard care. In this work, we used a mouse model of critical illness and recovery to study how muscle health deteriorates over a 28-day period. We found that different muscle groups wasted and then recovered at different rates depending on their fibre composition. The findings provide important insights into how muscles can recover from wasting and weakness caused by critical illness, with relevance to better treating the many patients experiencing muscle wasting and weakness after being in the ICU.

The [study] findings provide important insights into how muscles can recover from wasting and weakness caused by critical illness

**What are the potential implications of these results for disease biology and the possible impact on patients?**

The study findings have implications for better understanding the underlying causes of muscle dysfunction experienced by many patients in critical illness settings, especially factors that might interfere with recovery. The model provides an experimental platform for testing novel interventions to tackle critical illness and identify treatments that can promote recovery of muscle function and improve quality of life for patients.

**Figure:**
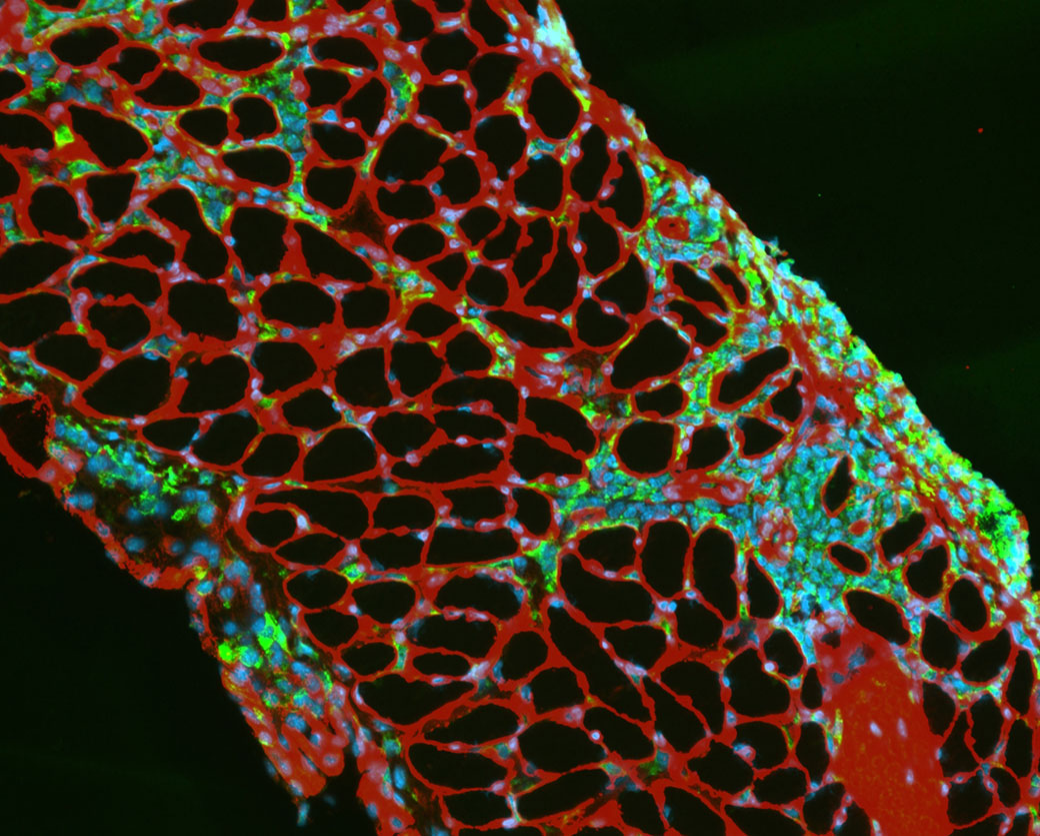
**Immunofluorescence assessment of the diaphragm muscle cross-section reveals infiltration of CD68^+^ immune cells (green) between individual muscle fibres (laminin; red), with nuclei shown in blue (DAPI), 14 days after zymosan-induced critical illness.** Image acquired by Amy Bongetti.

**Why did you choose DMM for your paper?**

We chose to submit this work to DMM due to the strong emphasis on developing new models of human disease. Our work is intended to be a resource for the field to model how critical illness affects skeletal muscle health. DMM provides an important platform for recognising and showcasing these new models that could advance understanding of human diseases.

**Given your current role, what challenges do you face and what changes could improve the professional lives of other scientists in this role?**

Funding security is an omnipresent challenge for early-career researchers. While passion drives the work for scientific inquiry, this cannot occur without secure funding to support the careers of aspiring academic researchers.

While passion drives the work for scientific inquiry, this cannot occur without secure funding to support the careers of aspiring academic researchers

**What's next for you?**

Currently, I am interested in the role of the immune system in regulating skeletal muscle homeostasis and how it contributes to muscle adaptation and plasticity. I also have a strong interest in women's health and preserving muscle function as we age.

**Tell us something interesting about yourself that wouldn't be on your CV**

Outside of academia, I am an avid reader and enjoy watching and playing sports.
